# Performance Evaluation of Miniature Integrated Electrochemical Cells Fabricated Using LTCC Technology †

**DOI:** 10.3390/s19061314

**Published:** 2019-03-15

**Authors:** Paulina Szymanowska, Damian Nowak, Tomasz Piasecki

**Affiliations:** Faculty of Microsystem Electronics and Photonics, Wrocław University of Technology, Z. Janiszewskiego 11/17, 50-372 Wrocław, Poland; damian.nowak@pwr.edu.pl (D.N.); tomasz.piasecki@pwr.edu.pl (T.P.)

**Keywords:** electrochemical impedance spectroscopy, cyclic voltammetry, protein adsorption, LTCC

## Abstract

Miniaturized and integrated analytical devices, including chemical sensors, are at the forefront of modern analytical chemistry. The construction of novel analytical tools takes advantage of contemporary micro- and nanotechnologies, as well as materials science and technology. Two electrochemical techniques were used in experiments: electrochemical impedance spectroscopy and cyclic voltammetry. The goal of this study was to investigate electron transfer resistance in a model solution containing ${\mathrm{Fe}}^{2+/3+}$ ions and protein adsorption using integrated electrochemical cells with different geometry. Tests performed at various ${\mathrm{Fe}}^{2+/3+}$ concentration allowed to verify that these cells work properly. The influence of bovine serum albumin adsorbing to the surface of the integrated electrochemical cells was investigated. In electrochemical impedance spectroscopy, the value of $R_{ct}$ increased with protein adsorption and the relative change of $R_{ct}$ was in range 21% to 55%. In cyclic voltammetry the decreasing amperometric response of the working electrode was used as evidence of protein adsorption on the electrode.

## 1. Introduction

Over the years, many methods were invented or used in the investigation of protein layers immobilized on the surface. The most commonly used are the techniques based on immunological and fluorescent tests. Presently the most popular method is Enzyme-Linked Immunosorbent Assay (ELISA) [1]. ELISA is a versatile and sensitive technique that can be used for qualitative or quantitative determinations of antigen or antibody [2,3]. The addition of substrate giving colored, fluorescent or luminescent reaction products makes it possible to determine the concentrations of the reactants at low levels [1]. The second type of methods are marker—free techniques which include optical (ellipsometry [4]), microscopic (atomic force microscopy (AFM)) [5], transmission electron microscopy (TEM) [6]) and electrical (potentiometric, voltammetric [7], impedance [7]).

Electrochemical impedance spectroscopy (EIS) and cyclic voltammetry (CV) are electrical techniques in which the electrochemical cells are used. They consist of three individual electrodes (working electrode—WE, reference electrode—RE and counter electrode—CE) placed inside the vessel. In EIS the electrical response of the investigated system to the small amplitude periodic small amplitude alternating voltage (AC) signal is measured [8]. The analysis of the system response provides information about the interface and reactions occurring in it. This technique is used in the investigation of material properties [7,9] and measurement of biological and biochemical layers, e.g., bacteria and proteins [10]. In cyclic voltammetry the current is measured as the potential is changed linearly with time, a current-potential curve is recorded. The resulting curve is called a linear potential sweep voltammogram or peak polarogram [11]. CV is proven to be very effective for the study of the following areas: the redox process on gold electrode [12], characterization of biomolecule-modified electrode surfaces and the analysis of the alteration in the interfacial properties originating from biomolecular recognition events [13].

Integration of the electrochemical cell electrodes on the surface of the common substrate allows for the cell miniaturization and facilitates the increase of the number of simultaneously conducted experiments in multiplexed measurement system. Integrated electrochemical cells can be fabricated on various substrates such as silicon [14], Low Temperature Co-fired Ceramic (LTCC) [15] and Printed Circuit Board (PCB) [16].

Integrated electrochemical cells (IEC) used in our measurements were fabricated using LTCC technology. The devices made using LTCC technology can be characterized by their chemical inactivity, hermeticity, high reliability and high temperature stability. This technology allows fabrication of microfluidic systems such as flow sensors, micropumps, microvalves, micromixers, microreactors and polymerase chain reaction (PCR) devices [17,18,19].

Electrochemical cells fabricated using LTCC technology were used in many chemical and biological measurements such as devices for the detection of cortisol [20], heavy metal detection system in biomedical fluids [21] and enzyme gas sensor [22]. For our experiments a multi-channel measurement system was created. It allows the detection of biological structures on eight LTCC electrochemical cells at the same time.

In this work, the preliminary experiments with the integrated electrochemical cells fabricated in the LTCC technology were conducted. The results of the test measurements with model solution containing ${\mathrm{Fe}}^{2+/3+}$ ions and the protein adsorption monitoring obtained using electrochemical impedance spectroscopy and cyclic voltammetry were shown.

## 2. Materials and Methods

**Figure 1:**
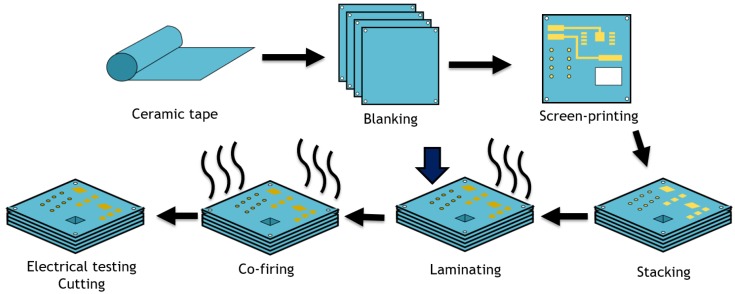
Schematic drawing of LTCC manufacturing process steps.

IEC were fabricated using LTCC technology. Schematic drawing depicting the LTCC manufacturing process steps is shown in Figure 1. In the first step of the manufacturing process the green tape was blanked and registration holes were made. In the next step, the conductors and passive components were deposited by a screen printing method. Finished sheets were stacked together and laminated. After this process the structures were co-fired in a furnace. Before or after the co-firing process, the LTCC structures were singulated using dicing saw, ultrasonic cutting or laser cutting.

**Figure 2:**
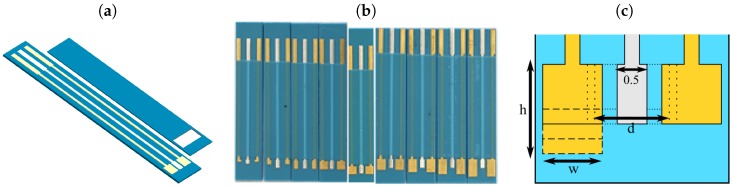
(**a**) Design of integrated electrochemical cell fabricated using LTCC technology; (**b**) Photograph of all types of integrated electrochemical cell fabricated using LTCC technology; (**c**) Schematic drawing presenting the integrated electrochemical cell dimensions.

The IECs used in the experiments presented in this paper were made on rectangular substrate (20 × 3.5 mm) and were formed using three layers of green tape (DP 951, DuPont—Stevenage, United Kingdom). Nine types of IECs were fabricated with electrodes varying in size (Figure 2) which dimensions are shown in Table 1. Sensors 1–4 differed in both the WE and CE length and the distance between the electrodes while in sensors 5–9 only the length of WE and CE changed. The width of the RE was 0.5 mm in all cases. Both gold working (WE) and counter electrodes (CE) as well as silver reference electrode (RE) and contact pads were screen-printed with 325 mesh stainless steel screen (conductive Au paste ESL 8880-H and Ag paste ESL 903A were used). After the lamination and shaping of structures, they were co-fired in chamber furnace with standard firing profile at maximum temperature 875 °C. The surface of fabricated IECs were investigated using optical and Scanning Electron Microscope (SEM). An Ag/AgCl reference electrode was formed using two methods: electrolysis [23] and immersion of an RE in a highly concentrated NaCl solution [24]. In EIS and CV experiments, one sensor of each type was used.

Fabricated IECs are meant to be used in the protein adsorption measurements done in the presence of the ${\mathrm{Fe}}^{2+/3+}$ ions. Those ions provided the occurence of the redox reaction on the electrodes surface [6]. Results presented in this work were obtained using model solution of potassium hexacyanoferrate (III) and potassium hexacyanoferrate (II) in either physiological saline or Phosphate Buffered Saline (PBS). Role of the latter was to maintain a constant pH. Providing constant measurement conditions is important because protein adsorption is a process with high sensitivity to the influence of the environment. The protein used in the experiment was Bovine Serum Albumin (BSA). BSA is a rigid, neutral, stable, moderately non-reactive protein [6,25]. It adsorbs to any type of surface and is highly stable [26].

### 2.1. Multichannel Electrochemical System EIS-STM32

**Figure 3:**
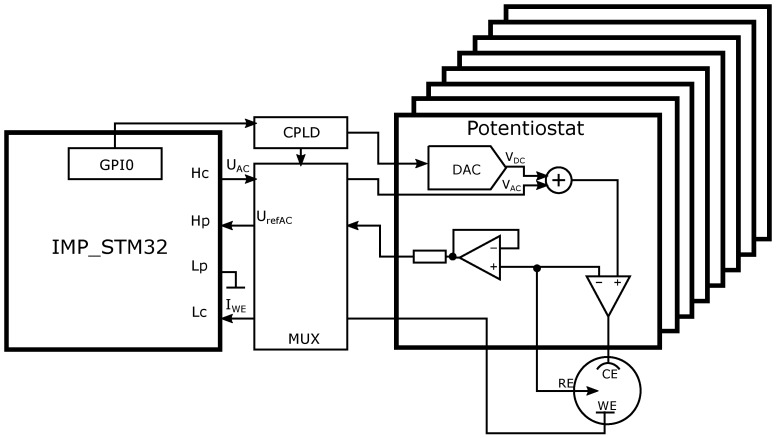
Schematic of eight-channel, compact, digitally controlled potentiostat dedicated for impedance analyzer IMP-STM32.

The eight-channel potentiostat of our own design was used in the measurements. It was designed as the EIS extension of the IMP-STM32 impedance analyzer [27]. Its simplified schematic is shown in Figure 3. The impedance analyzer is responsible for generating AC component of the excitation voltage and for WE current measurement (both AC and DC). The fast low-frequency impedance measurement algorithms implemented in the device allow to shorten the duration of the EIS spectra acquisition [28]. The General Purpose Input–Outtput (GPIO) port of the impedance analyzer interfaces with the potentiostat controlling its functions: channel switching using the analogue multiplexer (MUX), CE DC bias voltage generation and the separation of the RE voltage AC component.

**Figure 4:**
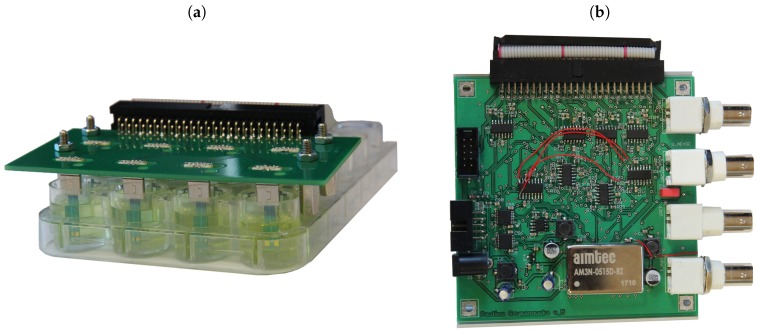
Measurement system: (**a**) eight-channel measurement head with sensors placed vertically in 24-well titrate plate and (**b**) eight-channel potentiostat.

Our system was designed to be fully compatible with the integrated electrochemical cells. IECs dimensions and contact pads placements were designed in such way that they fit into typical micro-USB connectors. The measurement head connected to the multichannel potentiostat has eight such connectors and may be placed on top of the titrate plate with sensors positioned vertically in eight of 24 wells. Impedance analyzer IMP-STM32 was used in the measurements and the range of the frequency sweep was from 1 Hz to 100 kHz. The measurement system is shown in the Figure 4. This system was controlled by a software which provided a multi-channel EIS and CV measurement. As a result EIS spectra and CV voltammograms were obtained.

### 2.2. Electric Equivalent Circuit Modeling

**Figure 5:**
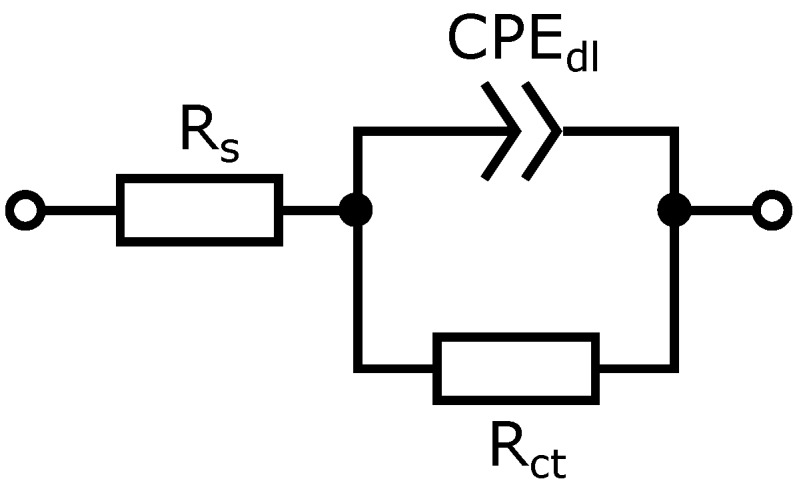
Electrical Equivalent Circuit used in experiment.

The EIS spectra were analyzed using the Electrical Equivalent Circuit (EEC) method with the circuit shown in Figure 5. The $R_{s}$ is the electrolyte resistance, $CPE_{dl}$ is the electrical double layer capacitance, $R_{ct}$ is the electron transfer resistance. A constant phase element (CPE) is used in modeling the behaviour of the imperfect RLC elements. The admittance of CPE is given by:

(1) $$Y=Q{\left(j\omega \right)}^{n}$$

where the *Q* (expressed in $Ss^{n}$) and *n* (dimensionless) are the parameters and ω is a radial frequency [8]. The influence of other factors such as electrodes and connections resistance on the $R_{s}$ was negligible. The preliminary tests revealed that they are smaller than 3 Ω which was less than 1% of $R_{s}$. Scribner ZView 3.2c software (Southern Pines, NC, USA) was used to obtain the values of EEC parameters from fitting of real spectra.

## 3. Results

### 3.1. Scanning Electron Microscope

**Figure 6:**
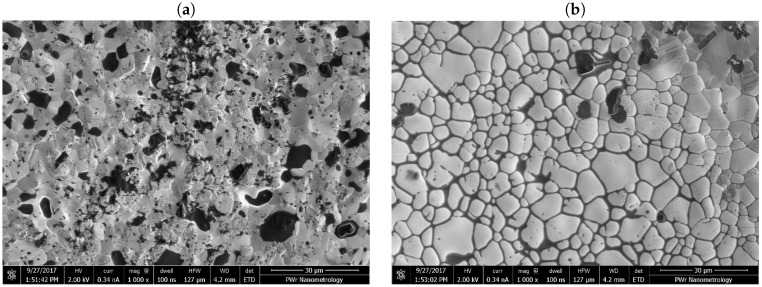
Electrodes surface of the integrated electrochemical cell on LTCC from SEM: (**a**) working electrode—gold and (**b**) reference electrode—silver.

The SEM images of the IEC electrodes are shown in Figure 6. As the electrodes were fabricated using LTCC technology the surface roughness was clearly visible. As the result the actual area of the WE may be larger than it would result from its planar dimensions. The dark spots in the SEM images were most probably the glassy remains of the paste used to screen-print the electrodes left after the sintering.

### 3.2. Reference Electrode Forming

**Figure 7:**
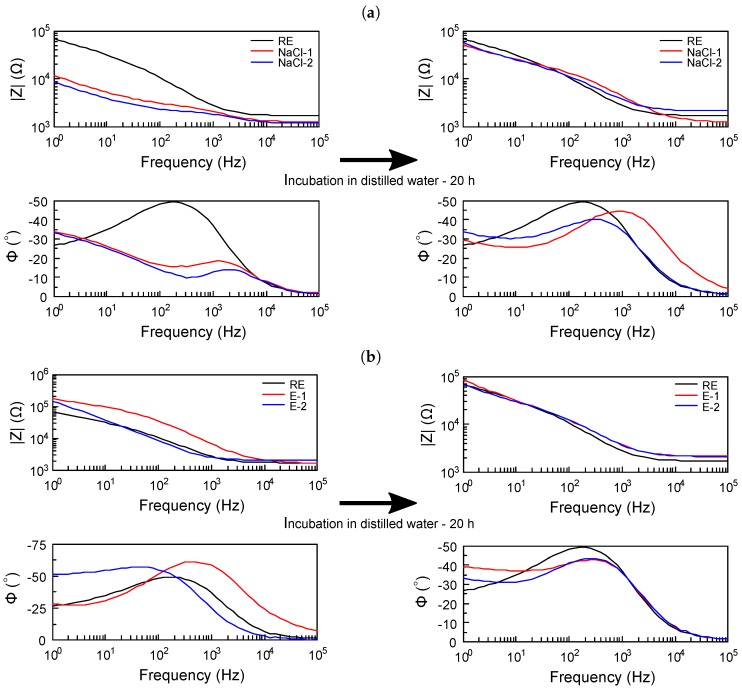
Bode plots for samples used as RE after (**a**) modification in NaCl solution and (**b**) electrolysis, where RE—commercial Ag/AgCl RE, NaCl-1 and NaCl-2—sample of Ag/AgCl RE formed in highly concentrated NaCl, E-1 and E-2—sample of Ag/AgCl RE formed using electrolysis.

The preliminary tests of the Ag/AgCl reference electrode on LTCC ceramic formed using both highly concentrated NaCl solution [24] and electrolysis [23] methods were carried out. Two samples were prepared with each method and marked as NaCl-1, NaCl-2 and E-1, E-2, respectively. After their forming the tested electrodes were used as a RE in traditional electrochemical cells and compared with commercial Ag/AgCl RE. On the Bode plots (Figure 7) the impedance spectra obtained using tested electrodes were compared. Measurements were performed twice: directly after RE forming and after keeping them in the distilled water for 20 h. Results obtained using both methods were similar. Immediately after RE forming they were not satisfactory indicating the necessity of their stabilization after which their performance was similar to the commercial RE. Simpler concentrated NaCl solution method was used for RE forming in further experiments.

**Figure 8:**
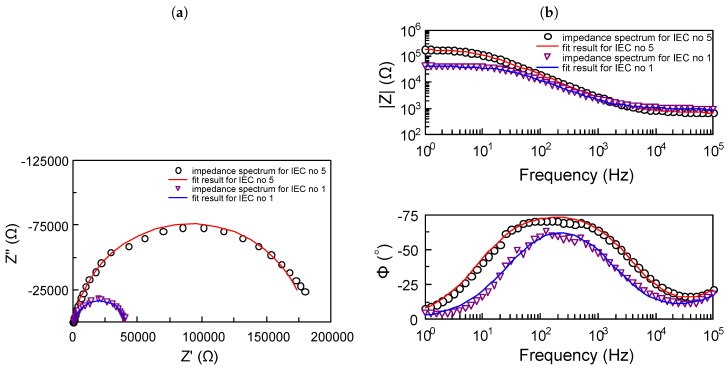
Impedance spectra and its approximation for sensors no 1 and 5 in PBS solution with the presence of ${\mathrm{Fe}}^{2+/3+}$ ions and BSA: (**a**) $Z^{\prime \prime }=f\left(Z^{\prime }\right)$, (**b**) |Z|=f(f) and Φ=f(f).

The Bode and Nyquist graphs (Figure 8—dots) show an example of the impedance spectra for complete IECs no 1 and 5 in a solution containing BSA. The spectra were approximated with electrical equivalent circuit (Figure 5). Results of the approximation are shown in Figure 8 as lines. The values of Chi-squared for this sensors were below 0.01.

### 3.3. Influence of the IEC Geometry on EIS Results

**Figure 9:**
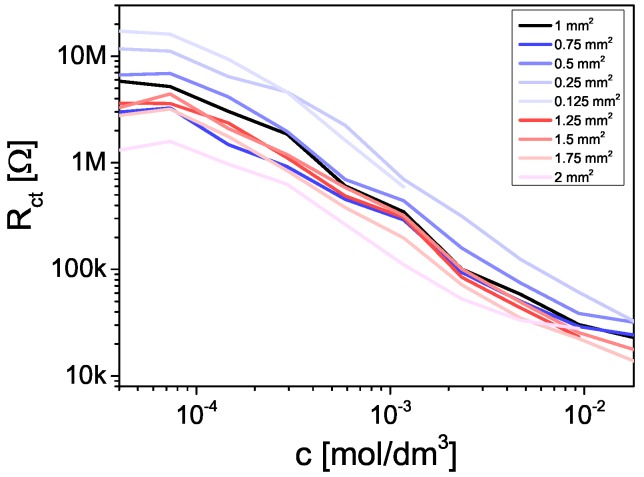
Charge transfer resistance as a function of the concentration of ${\mathrm{Fe}}^{2+/3+}$ ions for integrated electrochemical cell.

All types of the IECs were tested using EIS in the experiments which were carried out in the PBS solution with the presence of various concentrations of ${\mathrm{Fe}}^{2+/3+}$ ions. Obtained $R_{ct}$ was inversely proportional to the $c_{Fe^{2+/3+}}$ (Figure 9). It can be concluded that integrated sensors worked correctly. The obtained value of $R_{ct}$ was not greater than 1 MΩ at $c_{Fe^{2+/3+}}=1.17\frac{\mathrm{mmol}}{{\mathrm{dm}}^{3}}$, which is in the range which IMP-STM impedance analyzer is capable of precise measurement. It means that even the IEC with smallest electrodes may be easily used in further experiments conducted at similar $c_{Fe^{2+/3+}}$. Additionally it was concluded that further IECs miniaturization is still possible.

**Figure 10:**
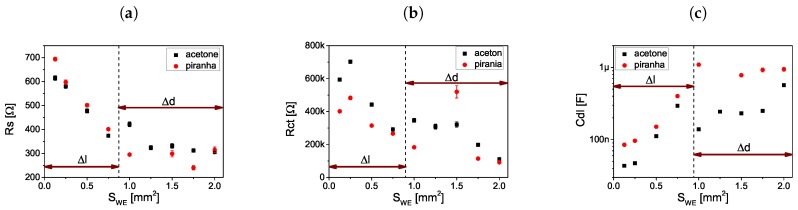
(**a**) Electrolyte resistance, (**b**) charge exchange resistance and (**c**) double layer capacitance as a function of the surface of the working electrode.

The influence of the electrode geometry on the components of the Equivalent Electrical Circuit (electrolyte resistance, electron transfer resistance and electrical double layer capacitance) is shown in Figure 10. Values of the parameters were constant for sensors 5–9 because the distance between the electrodes has not changed. For other sensors the value of the parameters varied with electrodes distance. IECs cell constants were calculated as well. Cell constant changed in the same way as the EEC parameters, cell constant varied with electrodes distance (1–5 Table 1) and remained about 2.7 cm${}^{-1}$ for other sensors.

### 3.4. EIS Monitoring of Protein Adsorption

**Figure 11:**
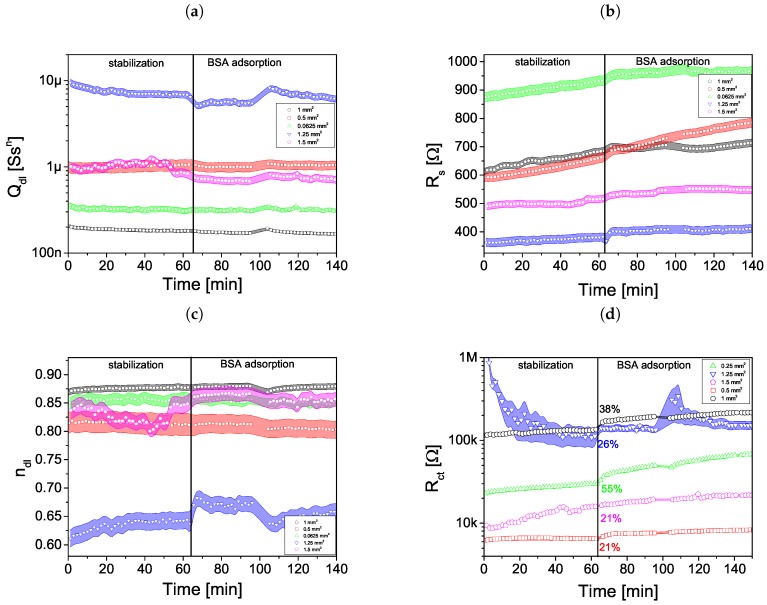
Parameters of electrical equivalent circuit as a function of time: (**a**) $Q_{dl}$, (**b**) $R_{s}$, (**c**) $n_{dl}$ and (**d**) normalized $R_{ct}$.

Protein adsorption to the WE surface was investigated using EIS for five type of sensors (Table 1 no 1, 3, 5–7). The experiment was divided into two steps. In the first step the impedance sensor was placed in 2 mL PBS solution with the presence of ${\mathrm{Fe}}^{2+/3+}$ ions ($c_{Fe^{2+/3+}}=5\frac{\mathrm{mmol}}{{\mathrm{dm}}^{3}}$) and stabilized for 60 min. Afterwards 10% solution was replaced by bovine albumin dissolved in buffer in the proportions of 0.1 mg BSA in 5 mL PBS. The time between the consecutive impedance spectra measurements was less than 2 min. Protein adsorption modified a solution-electrode system causing the change of EIS spectra and EEC parameters. The value of $R_{ct}$ gradually increased with protein adsorption on WE surface. The process lasted 70 min for sensor no 1 and 20–30 min for other sensors. At the end, the system was stabilized—parameters of EEC had a constant value. The values of the $R_{s}$, $Q_{dl}$ and $n_{dl}$ as a function of time are shown in Figure 11a–c.

It was observed that the obtained value of the electrical double layer capacitance $Q_{dl}$ did not correlate directly with the planar area of the electrodes. It was concluded that it was the effect of the WE surface roughness. As a result the attempt to normalize the $R_{ct}$ of each sensor to its planar dimensions would introduce significant error. Therefore to allow for easy comparison of the $R_{ct}$ between the sensors their $R_{ct}$ was scaled by the factor *k* calculated as:

(2) $$k=\frac{Q_{dl}}{Q_{dl_{1mm^{2}}}}$$

where the $Q_{dl}$ is the value obtained for the sensor and $Q_{dl_{1mm^{2}}}$ is the value for the sensor with 1 mm${}^{2}$ planar WE area. The normalized $R_{ct}$ as a function of time are shown in Figure 11d. The relative change of $R_{ct}$ after BSA adsorption is shown in Table 2.

### 3.5. CV Monitoring of Protein Adsorption

The CV experiments were conducted in two steps. In the first step the cyclic voltammograms for five type of sensors (Table 1 no 1, 3, 5–7) were measured for bare gold WE in PBS solution with the presence of ${\mathrm{Fe}}^{2+/3+}$ ions ($c_{Fe^{2+/3+}}=5\frac{\mathrm{mmol}}{{\mathrm{dm}}^{3}}$). In the second step the cyclic voltammograms were carried out after BSA adsorption on WE. The complete sweep time was 5 min and range of potential sweep was −0.6 V to 0.6 V.

**Figure 12:**
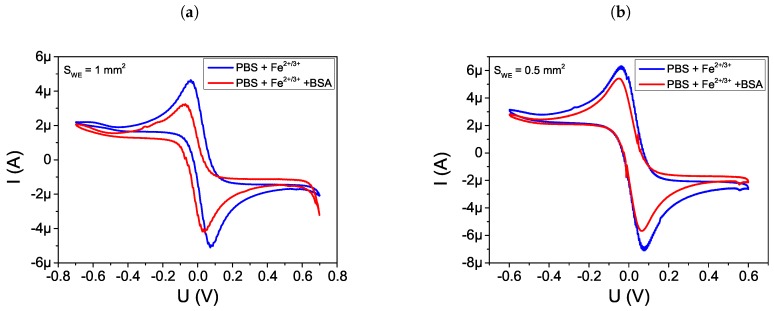

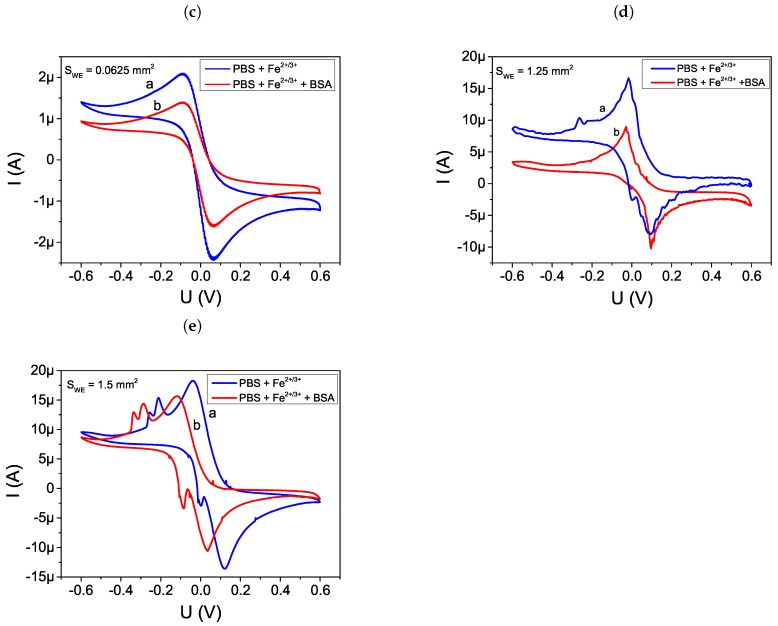
Cyclic voltammograms recorded in a 0.5 mM ${\mathrm{Fe}}^{2+/3+}$ + PBS solution after different step of modification, (a) bare Au WE, (b) BSA adsorption on WE electrode for sensors no (**a**) 5 ($S_{WE,CE}$ = 1 mm${}^{2}$), (**b**) 3 ($S_{WE,CE}$ = 0.5 mm${}^{2}$), (**c**) 1 ($S_{WE,CE}$ = 0.0625 mm${}^{2}$), (**d**) 6 ($S_{WE,CE}$ = 1.25 mm${}^{2}$) and (**e**) 7 ($S_{WE,CE}$ = 1.5 mm${}^{2}$).

Cyclic voltammetry is a simple and easy means of showing the changes of electrode behavior after each assemble step and CV experiments further confirmed that the BSA was successfully adsorbed on gold WE surface. The results of experiments of ${\mathrm{Fe}}^{2+/3+}$ ions at a bare gold WE (curve a) and WE after BSA adsorption (curve b) are shown in the Figure 12. Characteristics of diffusion-limited redox processes were observed at the bare WE. The decreasing amperometric response of the WE was used as evidence of protein adsorption on the electrode.

**Figure 13:**
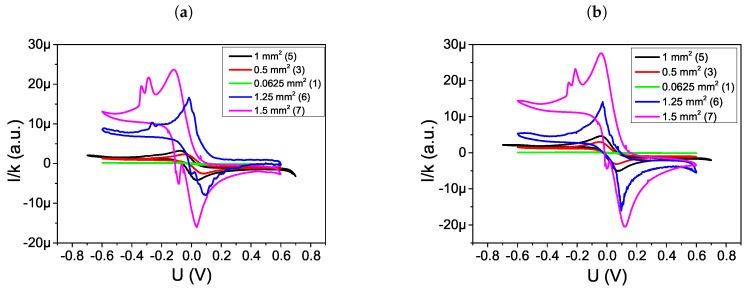
Normalized cyclic voltammograms recorded in a 0.5 mM ${\mathrm{Fe}}^{2+/3+}$ + PBS solution after different step of modification for every type of sensor, (**a**) bare Au WE, (**b**) BSA adsorption on WE electrode.

The normalized cyclic voltammograms of ${\mathrm{Fe}}^{2+/3+}$ ions at a bare gold WE and WE after BSA adsorption for all types of sensors are shown in the Figure 13. Measurement results were scaled by the factor *k* for the same reason as mentioned in Section 3.4. Decreasing the area of WE cause a reduction in the current value. Additional peaks at the sensors 6 and 7 may result from contamination or insufficient working electrode surface quality.

## 4. Conclusions

Miniature integrated electrochemical cells fabricated using LTCC technology were used in measurement with model solution containing ${\mathrm{Fe}}^{2+/3+}$ ions and for investigation of protein adsorption on working electrode. Tests performed at various ${\mathrm{Fe}}^{2+/3+}$ concentrations confirmed their proper operation. Electrodes geometry influenced cell constant and charge transfer resistance. Cell constant was in range of 2.5 to 5.1 cm${}^{-1}$. $R_{ct}$ determined for ${\mathrm{Fe}}^{2+/3+}$ concentration of $1.17\frac{\mathrm{mmol}}{{\mathrm{dm}}^{3}}$ was not greater than 1 MΩ. The influence of organic substances (BSA) adsorbing to the surface of the IECs was investigated by EIS and CV. The value of $R_{ct}$ increased with protein adsorption and the relative change of $R_{ct}$ was in the range of 21–55%. The largest relative $R_{ct}$ change after BSA adsorption (55%) was observed for the IEC with the smallest surface of working electrode (0.0625 mm${}^{2}$).

The results of the presented preliminary work confirmed that the IECs were used in the measurement with the presence of ${\mathrm{Fe}}^{2+/3+}$ ions and seem suitable for multichannel EIS systems for biological layer measurements. In the future, such sensors may also be integrated in complete microfluidic systems fabricated using LTCC technology.

## Figures and Tables

**Table 1:** Dimensions of the integrated electrochemical sensors and calculated values of $R_{ct}$ for $c_{Fe^{2+/3+}}$ = $1.17\frac{\mathrm{mmol}}{{\mathrm{dm}}^{3}}$ and cell constant, where *h*—height of WE and CE, *w*—width of WE and CE, *d*—distance between WE and CE, $A_{WE,CE}$—area of WE and CE, $A_{RE}$—area of RE, $R_{ct}$—charge transfer resistance, κ—cell constant.

No	*h* (mm)	*w* (mm)	*d* (mm)	$A_{\mathrm{WE},\mathrm{CE}}\;\left({\mathrm{mm}}^{2}\right)$	$A_{\mathrm{RE}}\;\left({\mathrm{mm}}^{2}\right)$	$R_{\mathrm{ct}}\;\left(k\Omega \right)$	$\kappa \;({\mathrm{cm}}^{-1})$
1	0.125	0.5	2	0.0625	0.125	594.2	5.1
2	0.5	0.5	1.5	0.25	0.5	702.5	4.8
3	1	0.5	1.5	0.5	0.5	442.1	3.9
4	0.75	1	1	0.75	0.5	291.7	3.1
5	1	1	1	1	0.5	346.7	3.5
6	1.25	1	1	1.25	0.5	321.4	2.7
7	1.5	1	1	1.5	0.5	308.5	2.7
8	1.75	1	1	1.75	0.5	197.5	2.6
9	2	1	1	2	0.5	110.8	2.5

**Table 2:** Relative change of $R_{ct}$ for every type of sensor.

No	$S_{\mathrm{WE}}$ (mm${}^{2}$)	Relative Change of $R_{ct}$
1	0.0625	55%
3	0.5	21%
5	1	38%
6	1.25	26%
7	1.5	21%

## Abbreviations

The following abbreviations are used in this manuscript:

AFM	atomic force microscopy
BSA	Bovine Serum Albumin
CE	counter electrode
CPE	constant phase element
CV	cyclic voltammetry
EEC	Electrical Equivalent Circuit
EIS	electrochemical impedance spectroscopy
ELISA	Enzyme-Linked Immunosorbent Assay
IEC	integrated electrochemical cell
LTCC	Low Temperature Cofired Ceramic
PBS	Phosphate Buffered Saline
PCR	polymerase chain reaction
RE	reference electrode
SEM	scanning electron microscope
TEM	transmission electron microscopy
WE	working electrode
